# Milk Production Performance, Handling and Utilization Practices, and Compositional Analysis of Local Goats in Garda Marta District, Southern Ethiopia

**DOI:** 10.1155/vmi/4334987

**Published:** 2026-05-25

**Authors:** Mardofe Manta, Seifu Birhanu, Yilkal Tadele

**Affiliations:** ^1^ Department of Animal Science, Arba Minch University, Arba Minch, Ethiopia, amu.edu.et

**Keywords:** chemical composition, goat, handling, milk yield, Southern Ethiopia

## Abstract

This study evaluated milk production performance, handling and utilization practices, and the compositional quality of local goats in Garda Marta district, Southern Ethiopia. A combination of household survey, on‐farm milk yield measurements, and laboratory‐based milk composition analysis was employed. A total of 138 households were selected using purposive and random sampling techniques to collect data on milking, handling, and utilization practices. For milk yield and composition analysis, 60 lactating does (15 per kebele) were purposively selected. Daily milk yield (DMY) was recorded from the 7th day postpartum until the end of lactation. Milk samples were collected at early, mid, and late lactation stages and analyzed for protein, fat, lactose, solid‐not‐fat (SNF), total solids (TSs), pH, and density. The results indicated that 97.8% of the households consume goat milk, with 55.1% consuming it in the raw form. Traditional containers such as gourds, clay pots, and plastic vessels are commonly used for milk handling and storage. About 58.7% of the households process milk into yoghurt. DMY differed significantly (*p* < 0.05) across lactation stages, with mean values of 0.79 ± 0.03, 0.85 ± 0.03, and 0.75 ± 0.03 L/day for early, mid, and late lactation, respectively. Lactation milk yield was significantly higher (*p* < 0.05) in lowland (59.60 L) than that in midland (53.27 L) areas. However, the milk composition was not significantly affected (*p* > 0.05) by agroecology or other factors. The study demonstrates that goat milk plays a vital role in household nutrition and livelihoods in the study area. Improving husbandry practices, milk handling, and extension services could enhance productivity and contribute to food security and rural development.

## 1. Introduction

Goat production is one of the most efficient low‐input livestock systems, as goats possess broad feeding habits, adaptability to unfavorable environmental conditions, and a short reproductive cycle [1, 2]. In Ethiopia, goats are kept for multiple purposes, including milk and meat production [3], income generation and capital accumulation [4], and provision of skin and manure [5]. The sector plays a significant role in supporting rural livelihoods and contributes to the achievement of Sustainable Development Goals (SDGs), particularly those related to food security and health [6, 7].

However, the purposes of keeping goats vary across locations due to economic, cultural, and ecological differences [8, 9]. Goat milk is an important dietary component in pastoral and agropastoral areas of Ethiopia, where a large proportion of the goat population is found [10]. It accounts for approximately 16.7% of the total milk consumed in the country [11], with a reported daily milk yield (DMY) of about 1.13 kg [12].

Goat milk is nutritionally valuable due to its rich composition of protein, fat, lactose, vitamins, and minerals [11, 13, 14]. It can be consumed fresh or processed into various products such as cheese, butter, yoghurt, and other dairy products [15]. Moreover, goat milk plays an important role in human nutrition, particularly for children, malnourished individuals, and people with cow milk allergies [16].

Assessing milk production performance, handling, utilization practices, and compositional quality of local goats is essential for improving productivity and ensuring food safety. Such studies also contribute to achieving SDGs, particularly SDG 2 (Zero Hunger), SDG 3 (Good Health and Well‐being), and SDG 13 (Climate Action).

Garda Marta district is one of the districts in Gamo Zone with a relatively large goat population (126,657) [17]. Despite this, there is limited information on milk production performance, handling practices, and milk composition in the area. Therefore, this study was initiated to evaluate milk production performance, assess milk handling and utilization practices, and analyze the compositional quality of local goat milk in Garda Marta district, Southern Ethiopia.

## 2. Materials and Methods

### 2.1. Study Area

Garda Marta is a district located in Gamo Zone, Southern Ethiopia, at approximately 5°89′N latitude and 37°07′E longitude, covering about 46787.79 ha of land. The total human population of the district is estimated at 88,267 [17]. The altitude ranges from 1000 to 1200 m above the sea level. The district receives an annual rainfall of 1200–1600 mm and experiences mean annual temperatures ranging from 25°C to 28°C. The agroecology of the district is characterized by 30% midland and 70% lowland areas. The dominant farming system is a mixed crop–livestock production system. Major crops grown in the area include maize, sorghum, teff, haricot bean, coffee, and enset (*Ensete ventricosum*).

The livestock population of the district is estimated at 169,370 cattle, 126,657 goats, 65,790 sheep, 14,742 donkeys, 651 horses, 949 mules, 207,395 chickens, and 102,597 beehives [17, 18]. The study was conducted in Laysha kebele, representing the midland agroecology, and in Shakaro, Gogale, and Lado kebeles, representing the lowland agroecology.

### 2.2. Sampling and Sample Size

The study consisted of a household survey, field measurements, and milk composition analysis. For the survey, multistage purposive and random sampling techniques were employed to select study kebeles (the smallest administrative units in Ethiopia) and respondent households.

In the first stage, the district was stratified into two agroecological zones: midland and lowland. In the second stage, study kebeles were purposively selected based on road accessibility, goat population size, and representation of the agroecological zones. In the third stage, households rearing goats in each selected kebele were identified and listed. Finally, respondent households were selected randomly from the prepared household lists. The total sample size was determined by using the Cochran formula [19]: *n*_*o*_ = *Z* ^2^∗(*P*)(*q*)/*e* ^2^, where *n*_*o*_ = desired sample size, *Z* = standard normal deviation (1.96 for 95% confidence level), *P* = 0.1 (proportion of population to be included in sample), *q* = 1−0.1, i.e., (0.9), and *e* = desired level of precision (margin of error, e.g., 0.05 for 5%).

Based on the sample size determination, a total of 138 households were selected from 1380 goat‐rearing households in the study kebeles. The sample was proportionally allocated across the kebeles according to the number of goat‐rearing households, resulting in 39 households from Laysha (midland), 17 from Lado (lowland), 39 from Shakaro (lowland), and 43 from Gogale (lowland). This ensured adequate representation of households across the different agroecological zones.

For the evaluation of milk yield and composition, a total of 60 lactating does (15 from each study kebele) were purposively selected to ensure that only healthy animals with comparable lactation stage and parity were included. Milk samples were collected starting from 7 days after kidding to allow kids to suckle colostrum from their dams.

### 2.3. Methods of Data Collection

Primary data were collected using a questionnaire survey, field measurements, and laboratory analysis of milk composition.

#### 2.3.1. Household Survey

Experts from the Livestock and Fishery Resources Development Office who are familiar with the local language were recruited and trained on the administration of the questionnaire. The questionnaire was initially prepared in English and then translated into the local language. A structured questionnaire interview was employed to collect data on the socioeconomic characteristics of households, goat milking and milk handling practices, milk processing, milk utilization practices, and associated constraints.

#### 2.3.2. Milk Yield Measurement

DMY was recorded on‐farm starting from the 7th day after kidding until the end of lactation by trained livestock development experts in each kebele under close supervision of the researchers. Milk samples were collected at three stages of lactation: early, mid, and late. The lactating does were categorized based on days in milk as early (34–49 days postkidding; *n* = 20), mid (53–70 days postkidding; *n* = 20), and late (87–137 days postkidding; *n* = 20).

Milk was collected from manually milked does and measured for each animal. Sampling was conducted twice daily (morning at 06:00 h and evening at 18:00 h). Milk yield was measured using a graduated measuring cylinder, and readings were taken at the lower meniscus during each milking. Morning and evening milk yields (liters/doe) were recorded, and total DMY was calculated accordingly.

The weigh–suckle–weigh method was used to estimate the amount of milk consumed by kids [20]. The total DMY was calculated as the sum of milk obtained through hand milking and the amount suckled by the kids during both morning and evening sessions. Lactation length (LL) was determined by recording the kidding date of each doe and subtracting it from the date of data collection.

### 2.4. Milk Composition Analysis

Approximately 20 mL of milk was collected from each goat in a 50 mL plastic bottle twice per week and stored in a cool box until analysis. Samples were analyzed shortly after collection (within 1‐2 h) using a Lacto Scan SP/60 analyzer (Model 328,617, Bulgaria). A total of 240 milk samples were obtained from the 60 lactating goats. Each sample was analyzed for protein, fat, lactose, solid‐not‐fat (SNF), total solids (TSs), pH, and density.

### 2.5. Data Analysis

All collected data were checked for errors that may have occurred during data collection. Descriptive statistics were used to summarize and describe categorical variables. The chi‐square test was employed to examine significant associations among categorical variables and agro‐ecological zones. Quantitative variables were analyzed using the General Linear Model (GLM) procedure (PROC GLM) of SAS Version 9.2 [21]. Differences in milk yield and composition between two groups (e.g., agroecology: midland vs. lowland and birth type: single vs. twin) were analyzed using the independent samples *t*‐test. For variables with three or more groups (e.g., age of doe, lactation stage, and parity), one‐way analysis of variance (ANOVA) was conducted. Where significant differences were observed, mean separation was performed using Tukey’s test, and statistical significance was declared at *p* < 0.05.

Two different models were used. The first model to analyze milk yield and composition between two groups was *Y*_*i* *j*_ = *μ* + *α*_*i*_ + *ɛ*_*i* *j*_, where *Y*_*i* *j*_ = the response variable, *μ* = overall mean, *α*_*i*_ = fixed effect of agro ecology (*i* = mid land and *ii* = highland) or birth type (single, twin), and *ɛ*_*i* *j*_ = residual error.

The second model to analyze variables with three or more groups (age of doe, lactation stage, and parity) was *Y*_*i* *j* *l*_ = *μ* + *A*_*i*_ + *L*_*j*_ + *P*_*l*_ + *ε*_*i* *j* *l*_, where: *Y*_*i* *j* *l*_ = milk yield or milk composition, *μ* = overall mean; *A*_*i*_ = fixed effect of the *i*th age of doe (*i* = 1, 2, 3, 4, and 5), *L*_*j*_ = fixed effect of the *j*th lactation stage (*j* = early, mid, and late), *P*_*l*_ = fixed effect of *l*th parity (*l* = 1, 2, 3, 4, 5, and ≥ 6), and *ε*_*i* *j* *l*_ = random error.

## 3. Results

### 3.1. Milk Yield of Goats

The milk yield and LL of goats in the study area are presented in Table 1. Mean DMY and total lactation yield differed significantly (*p* < 0.05) between agroecologies, with goats in the lowlands producing higher yields than those in the midlands. Milk yield also varied significantly (*p* < 0.05) with the birth type, where does that kidded twins produced more milk than those with a single kid. Lactation stage had a significant effect (*p* < 0.05) on DMY, with the midlactation stage yielding higher milk than early and late stages. No significant differences (*p* > 0.05) were observed in DMY among different age groups or parities of does.

**TABLE 1:** Mean DMY, LMY, and LL of local goats.

Variables	*N*	DMY (L)	LMY (L)	LL (days)
Agro‐ecology				
Midland	15	0.75^b^	53.27^b^	71.77
Lowland	45	0.84^a^	59.60^a^	71.37
*p* value		0.0080	0.0272	0.5101
Birth type				
Single	40	0.64^b^	47.53^b^	75.75^a^
Twins	20	0.95^a^	65.35^a^	67.39^b^
*p* value		< 0.0001	< 0.0001	0.0100
Age of the doe				
1 year	14	0.75	55.3	77.96
2 years	22	0.79	52.9	66.69
3 years	21	0.84	60.54	72.2
4 years	3	0.79	57.03	69.42
*p* value		0.4853	0.2279	0.5101
Lactation stage				
Early	20	0.79^ab^	34.56^c^	
Mid	20	0.853^a^	53.24^b^	
Late	20	0.75^b^	81.52^a^	
*p* value		0.0160	< 0.0001	
Parity				
1	14	0.80	53.59	66.18
2	2	0.87	58.90	67.78
3	19	0.84	60.54	72.81
4	13	0.83	59.19	71.89
5	9	0.74	51.44	73.00
6	3	0.68	54.97	77.77
*p* value		0.4853	0.2279	0.4858

*Note:* Means within the column with different superscript are significant different at *p* < 0.05, *N* = number of milking goats.

Abbreviations: DMY, daily milk yield; LL, lactation length; LMY, lactation milk yield.

### 3.2. Utilization of Goat Milk in the Study Area

Almost all households (97.8%) in the study area reported consuming goat milk, while 2.2% did not (Table 2). Low milk yield from local goats and lack of awareness were cited as the main reasons for limited milk consumption. About half of the respondents (50%) indicated that children are primary consumers of goat milk.

**TABLE 2:** Goat milk utilization practices in the study area.

Parameter	Agro‐ecology		Overall *N* (%)	*X* ^2^	*p* value
	Midland *N* (%)	Lowland *N* (%)			
Do you consume goat milk?					
Yes	39 (100.0)	96 (97.0)	135 (97.8)	12.22	0.01
No	0 (0.0)	3 (3.0)	3 (2.2)		
Reasons for less goat milk consumption					
Low milk yield of local goats	16 (41.1)	59 (59.6)	75 (54.4)	10.91	0.012
Lack of awareness	23 (58.9)	24 (40.4)	34 (45.6)		
Goat milk utilizers					
Nursing mothers	2 (5.1)	6 (6.1)	8 (5.8)	9.49	0.024
Children	23 (59)	46 (46.5)	69 (50)		
Adults/aged people	9 (23.1)	33 (33.3)	42 (30.4)		
Sick individuals	3 (7.7)	7 (7.1)	10 (7.3)		
Pregnant mothers	2 (5.1)	4 (4)	6 (4.3)		
How milk is consumed?					
Raw milk	20 (51.3)	56 (56.6)	76 (55.1)	11.2	0.004
After souring	9 (23.1)	15 (15.2)	24 (17.4)		
After boiling	10 (25.6)	25 (25.2)	35 (25.3)		

*Note: N* = number of respondents.

### 3.3. Milking and Milk Handling Practices

All households (100%) in the study area reported using clean milking equipment (Table 3). Water, detergent, and tree leaves were commonly used for cleaning. A majority of households (63.7%) wash their hands and milking utensils before milking. Most respondents (81.2%) use gourds (dried fruit shells) for milk storage, while 15.9% use plastic containers and 2.9% use clay pots.

**TABLE 3:** Traditional dairy goat milking and handling practices in the study area.

Variables	Agroecology		Overall *N* (%)	*X* ^2^	*p* value
	Midland *N* (%)	Lowland *N* (%)			
Milking frequency					
Once/day	0 (0)	18 (18.2)	18 (13)	13.97 0.001	
Twice/day	39 (100)	81 (81.8)	120 (87)		
Do you clean milking and milk utensils?					
Yes	39 (100)	99 (100)	138 (100)		
How do you clean the equipment?					
Washing with water and detergent	11 (28.2)	28 (28.3)	39 (28.3)	10.72	0.032
Washing with water and tree leaves	23 (59)	56 (56.6)	79 (57.2)		
Washing with water, detergent and tree leaves	5 (12.8)	15 (15.1)	20 (14.5)		
How do you perform hygienic practices?					
Wash milker hands and milking utensils	22 (56.4)	66 (66.7)	88 (63.7)	9.87	0.041
Washing udder before milking	14 (35.9)	25 (25.2)	39 (28.3)		
Washing udder after milking	3 (7.7)	8 (8.1)	11 (8)		
Materials used for milk storing					
Clay pot	4 (10.2)	0 (0)	4 (2.9)	11.21	0.02
Gourd	34 (87.2)	78 (78.8)	112 (81.2)		
Plastic material	1 (2.6)	21 (21.2)	22 (15.9)		

*Note: N* = number of respondents.

### 3.4. Yoghurt Making Practice

The majority of households (58.7%) in the study area produce yoghurt from goat milk (Table 4). About 33% of households allow the milk to ferment for 3 days, while 23% let it ferment for 2 days.

**TABLE 4:** Yoghurt making practice in the study area.

Variables	Agroecology		Overall *N* (%)	*X* ^2^	*p* value
	Midland *N* (%)	Lowland *N* (%)			
Do you process goat milk?					
Yes	23 (59)	58 (58.6)	81 (58.7)	4.12	0.53
No	16 (41)	41 (41.4)	57 (41.3)		
What product do you process?					
Yoghurt	23 (59)	58 (58.6)	81 (58.7)		
Do you make yoghurt?					
Yes	23 (59)	58 (58.6)	81 (58.7)	5.46	0.13
No	16 (41)	41 (41.4)	57 (41.3)		
How long time do you store milk for yoghurt making?					
2 days	10 (25.6)	22 (22.2)	32 (23.2)	10.30	0.035
3 days	11 (28.2)	35 (35.4)	46 (33.3)		
4 days	2 (5.2)	1 (1.0)	3 (2.2)		
Yoghurt storing material					
Gourd	17 (43.6)	52 (52.5)	69 (50)	9.78	0.047
Plastic material	6 (15.4)	6 (6.1)	12 (8.7)		

*Note: N* = number of respondents.

### 3.5. Milk Composition of Dairy Goats

The milk composition of dairy goats in the study area, including protein, fat, lactose, SNF, TSs, pH, and milk density, is presented in Table 5. There were no significant effects (*p* > 0.05) of agroecology, birth type, age, lactation stage, or parity on any of the milk composition traits.

**TABLE 5:** Milk composition (% DM) of local goats in the study area.

Variable	*N*	Protein	Fat	Lactose	SNF	TS	pH	Density
Agroecology								
Midland	15	4.57	5.43	4.66	9.97	15.37	6.73	1035.8
Lowland	45	4.73	6.18	4.71	9.96	16.18	6.64	1032.6
*p*‐value		0.3401	0.1256	0.7395	0.9631	0.1499	0.2256	0.0563
Birth type								
Single	40	4.77	6.17	4.59	9.68	15.89	6.69	1033.7
Twins	20	4.53	5.44	4.77	10.25	15.66	6.69	1034.7
*p* value		0.1071	0.1017	0.2145	0.1059	0.6659	0.8770	0.4981
Age of the doe								
1 year	14	4.11	6.15	4.30	10.40	16.56	6.65	1034.1
2 years	22	4.95	6.19	4.67	9.56	15.7	6.7	1032.2
3 years	21	4.83	5.43	4.74	10	15.43	6.7	1034.4
4 years	3	4.71	5.44	5.02	9.91	15.39	6.71	1036.2
*p* value		0.2744	0.6992	0.5551	0.7911	0.8677	0.9782	0.7556
Lactation stage								
Early	20	4.53	6.10	4.49	9.62	15.68	6.70	1032
Mid	20	4.64	5.35	4.77	10.28	15.63	6.72	1035.2
Late	20	4.78	5.96	4.78	10.01	16.02	6.65	1035.5
*p* value		0.3053	0.2880	0.1086	0.2515	0.7542	0.2466	0.0751
Parity								
1	14	5.18	4.95	5.08	10.31	15.29	6.65	1034.5
2	2	5.23	6.4	5.27	9.5	15.93	6.77	1034.8
3	19	4.31	5.82	4.55	10.48	16.32	6.7	1036.5
4	13	4.43	5.28	4.75	10.14	15.4	6.63	1034.5
5	9	4.48	5.7	4.53	10.1	15.77	6.71	1033.6
6	3	4.26	6.69	3.92	9.26	15.94	6.68	1031.6
*p* value		0.2877	0.6159	0.0762	0.6678	0.8948	0.5057	0.8712

*Note: N* = number of milking goats.

Abbreviations: SNF, solid‐not‐fat; TS, total solids.

## 4. Discussion

Variation in milk yield among goats may result from differences in management, environmental conditions, and mammary gland activity [22]. Matebesi et al. [23] reported that lowland goats in Lesotho produced more milk (0.42 L/day) than mountain goats (0.32 L/day). Comparable yields include 0.85 ± 0.12 L/day for Somali goats [12] and 0.75 L/day for Begait goats [9]. Lower yields were reported for Hararghe highland goats (0.40 L/day; [24]), Somali goats (0.53 L/day; [25]), and Central highland goats (0.34 ± 0.02 L/day; [26]). DMY in this study was lower than 1.13 ± 0.13 L/day reported for Arsi‐Bale goats [12]. LL was also shorter than Begait (111 days; [9]), Hararghe highland (108 days; [24]), and Central highland goats (104.2 ± 4.45 days; [26]).

Does with twin kids produced more milk than single‐kidded does, consistent with Dhaba et al. [4], likely due to enhanced mammary development and suckling stimulation [27]. Milk yield was highest at midlactation [28] but differs from Norris et al. [29], who reported higher early‐stage yields. Parity had no significant effect, aligning with Norris et al. [29].

Goat milk is consumed by all family members, mainly children, though consumption varies due to low awareness and cultural taboos [30, 31]. Similar low consumption was reported in Humbo district (96.2%; [31]) and child‐focused consumption in Hadiya zone (88%; [32]). Most households consumed milk raw (55.1%), while others boiled (25.3%) or soured it (17.4%), consistent with 60% raw consumption in Kolla Tembien, Tigray [33].

Hygiene practices varied: 31%–35% of the households washed hands before milking in Karat Zuria and Uba Debra Tsehay [34], with no udder washing. Milk container cleaning ranged from 43.3% in pastoralists to 76.67% in agropastoralists in Degahbur [35]. In the study area, 87% milked goats twice daily, similar to Alefe [25]. Milk storage included gourd, plastic, and stainless steel [36]. Yogurt was naturally fermented without starter culture [37], with 7.69%–8.70% of the households preparing it in Karat Zuria and Uba Debra Tsehay [34].

Milk composition for Arsi‐Bale goats under semi‐intensive management included protein 4.8 ± 0.14%, fat 5.15 ± 0.04%, TS 16.27 ± 0.76%, and lactose 4.93 ± 0.11% [12]. Fat content of 5.3% was reported by Andualem et al. [28], while Nurfeta et al. [38] reported protein 3.5 ± 0.12%, fat 4.2 ± 0.17%, and TS 13.4 ± 0.15% under extensive management. Higher values were reported for Abergelle goats under semi‐intensive management (fat 8.71 ± 0.36% and TS 18.6 ± 0.45%; [39]). Milk pH ranged from 6.62 ± 0.01 to 6.64 ± 0.01 in Hair and Sannen × Hair goats [40]. Lactation stage affected lactose, increasing from early to late lactation [41], while parity had no effect on milk fat or protein [42].

The findings of this study highlight the significant contribution of local goat milk production and consumption to household nutrition and livelihoods, directly supporting the achievement of SDGs, particularly SDG 2 (Zero Hunger) through enhanced food security and SDG 3 (Good Health and Well‐being) by providing nutrient‐rich dairy for vulnerable populations.

## 5. Conclusions

Goat milk is widely consumed and supports household nutrition. Milk yield is influenced by agroecology, birth type, and lactation stage, with higher yields in lowlands and twin‐kidded does. Milk composition was not significantly affected by evaluated factors. Low milk yield and shorter LL indicate the need for improved feeding and management. Enhancing milk handling, husbandry practices, and extension services could improve productivity and food security.

## Author Contributions

Mardofe Manta: investigation, methodology, and writing–original draft preparation; Yilkal Tadele and Seifu Birhanu: analysis, supervision, resources, and writing–review and editing.

## Funding

No external funding was received for this study.

## Disclosure

All authors have approved the manuscript.

## Consent

Written informed consent obtained from all participants.

## Conflicts of Interest

The authors declare no conflicts of interest.

## Data Availability

Data used to support the findings of this study are available from the corresponding author upon reasonable request.
